# Virulence and Inoculation Route Influence the Consequences of *Mycoplasma hyorhinis* Infection in Bama Miniature Pigs

**DOI:** 10.1128/spectrum.02493-21

**Published:** 2022-04-21

**Authors:** Jia Wang, Lizhong Hua, Yuan Gan, Ting Yuan, Long Li, Yanfei Yu, Qingyun Xie, Ademola O. Olaniran, Thamsanqa E. Chiliza, Guoqing Shao, Zhixin Feng, Bala Pillay, Qiyan Xiong

**Affiliations:** a Institute of Veterinary Medicine, Jiangsu Academy of Agricultural Sciences, Key Laboratory of Veterinary Biological Engineering and Technology, Ministry of Agriculture and Rural Affairs, Nanjing, China; b College of Agriculture, Engineering & Science, University of KwaZulu-Natal, Durban, South Africa; c Xicheng Animal Husbandry and Veterinary Station, Jintan District, Changzhou, China; Innovations Thérapeutiques et Résistances

**Keywords:** *Mycoplasma hyorhinis*, virulence, inoculation route

## Abstract

Mycoplasma hyorhinis is a widespread pathogen in pig farms worldwide. Although the majority of *M. hyorhinis*-colonized pigs have no apparent clinical disease, the pathogen can induce diseases such as polyserositis, arthritis, and eustachitis in some cases. To explore the mechanisms for the occurrence of these diseases, we challenged 4 groups of Bama miniature pigs with *M. hyorhinis* isolated from pigs without clinical symptoms (non-clinical origin [NCO] strain) or with typical clinical symptoms (clinical origin [CO] strain) and investigated the impacts of different strains and inoculation routes (intranasal [IN], intravenous [IV] + intraperitoneal [IP], and IV+IP+IN) on disease induction. Another group of pigs was set as a negative control. Pigs inoculated with the CO strain through a combined intravenous and intraperitoneal (IV+IP) route showed a significant decrease in average daily weight gain (ADWG), serious joint swelling, and lameness compared with the pigs in the negative-control group. Furthermore, this group developed moderate-to-severe pericarditis, pleuritis, peritonitis, and arthritis, as well as high levels of IgG and IgM antibodies. Pigs inoculated IV+IP with the NCO strain developed less marked clinical, pathological changes and a weaker specific antibody response compared with the pigs inoculated with the CO strain. The challenging results of the NCO strain via different routes (IV+IP, IV+IP+IN, and IN) indicated that the combined route (IV+IP) induced the most serious disease compared to the other inoculation routes. Intranasal inoculation induced a smaller decrease in ADWG without obvious polyserositis or arthritis. These data suggest that differences in both strain virulence and inoculation route affect the consequences of *M. hyorhinis* infection.

**IMPORTANCE** Mycoplasma hyorhinis is a widespread pathogen in pig farms worldwide. The mechanisms or conditions that lead to the occurrence of disease in *M. hyorhinis*-infected pigs are still unknown. The objective of this study was to evaluate the impact of differences in the virulence of strain and the inoculation route on the consequences of *M. hyorhinis* infection.

## INTRODUCTION

Mycoplasma hyorhinis is ubiquitous in the pig population, frequently colonizing the tonsils and respiratory epithelium of the nasal cavity and the conducting airways. Understanding of the epidemiology of *M. hyorhinis* is still very poor due to its relatively recent recognition as an economically significant swine pathogen. Different studies have revealed that *M. hyorhinis* is often detected in older animals such as fattening pigs (1, 2). Piglets are colonized via their mother, sows, or older pigs in the herd. *M. hyorhinis* can be shed by nasal secretions and transmitted by direct contact between infected and naive pigs (3). The prevalence of *M. hyorhinis* differs between countries and herds but is generally high. For example, *M. hyorhinis* was detected in oral fluids in 97% of sampled herds in the United States (4) and up to 98% of animals post-weaning tested positive by PCR in another study (1). In Switzerland, 10% of lungs from animals with pneumonia tested positive, while in Germany the prevalence was 18.5% (2).

The majority of *M. hyorhinis*-colonized pigs have no apparent clinical disease. Therefore, *M. hyorhinis* was considered to be part of the bacterial community in the nasal cavity of piglets during their early life. However, subsequent clinical and scientific studies have confirmed the pathogenicity of *M. hyorhinis*. It has been confirmed that *M. hyorhinis* can induce conditions such as pleuritis, peritonitis, pericarditis, arthritis, and eustachitis (3), although the exact mechanisms or conditions that lead to the occurrence of disease are unknown. It is assumed that systemic dissemination is critical in the process of *M. hyorhinis-*associated diseases (3). However, the mechanisms leading to systemic spread are unclear. According to our current knowledge, coinfection by other pathogens (5, 6), stress (7), and hijacking of the host plasminogen/plasmin system (8) may play roles in the dissemination of *M. hyorhinis* from the original colonization site to other loci. The high prevalence of *M. hyorhinis* represents a serious concern, not only due to the disease itself, but also to the increased risk of more severe diseases and economic losses as a result of coinfection with other pathogens. Thus, a comprehensive understanding of the mechanisms of *M. hyorhinis* infections is critical for developing guiding principles to control the diseases it may cause.

The virulence of a strain is often the key factor in determining its disease-causing potential. This is true for many mycoplasma species. For example, there are numerous high-virulence strains of Mycoplasma hyopneumoniae (e.g., strain 232), as well as low-virulence and non-virulent strains (e.g., strains J and 168L, respectively) (9, 10). *In vivo* infection with different *M. hyorhinis* strains has been reported (11, 12); however, the differences in the virulence of *M. hyorhinis* strains and their impact on clinical consequences in infected pigs is still not clear.

In this study, we isolated two strains of *M. hyorhinis*: one from a pig with no clinical symptoms (asymptomatic) and another from a pig with typical clinical symptoms (symptomatic). These strains were used in challenge experiments to evaluate differences in clinical symptoms and pathological changes in infected pigs. We also explored the influence of infection routes on the disease by using different methods to inoculate pigs with the same strain.

## RESULTS

### Mycoplasma isolation.

**FIG 1:**
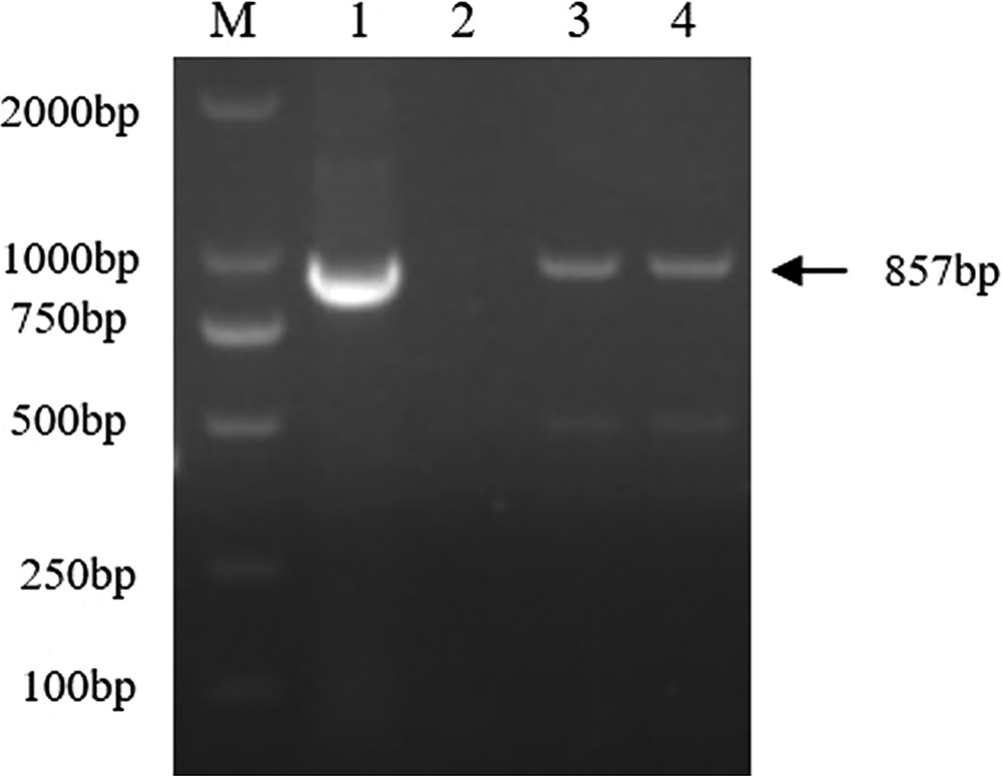
Identification of *M. hyorhinis* isolates. Identification by *M. hyorhinis* by specific PCR targeting the *p37* gene (857 bp). M, DNA marker; lane 1, positive control; lane 2, negative control; lanes 3 and 4, strains isolated from the asymptomatic and symptomatic pigs.

One pig with swollen joints and severe diffuse fibrinous peritonitis, from a farm with a known history of problems with *M. hyorhinis*-induced polyserositis and arthritis, was used for *M. hyorhinis* isolation. Tissues of the tonsil, lung, heart, and joint (swabs) were sampled. Another clinically healthy pig from a farm with very stable animal health, but which was positive for *M. hyorhinis* infection, was also sampled for pathogen isolation. None of the cultures with color changes appeared turbid. The pathogens isolated were all identified as *M. hyorhinis* by specific PCR targeting *p37* (Fig. 1). *M. hyorhinis* was isolated from the tonsil, lung, and heart tissues of the pig with clinical symptoms. However, *M. hyorhinis* was only isolated from the tonsil, and not from the other tissues sampled, in the healthy pig. The ST type of the strain isolated from the asymptomatic pig was identified as ST 13, which was denoted as a strain of non-clinical origin (NCO). The STs of the strains isolated from different tissues of the symptomatic pig were found to be the same, indicating a single strain, which was denoted as a strain of clinical origin (CO). Exact matches for all loci were not available in the PubMLST database; novel sequences were submitted for new allelic profiles and a new ST number (ST144) was assigned to the respective allele combinations. The CO strain isolated from the lung was used for the subsequent experiments.

### Clinical examination after bacterial challenge.

**FIG 2:**
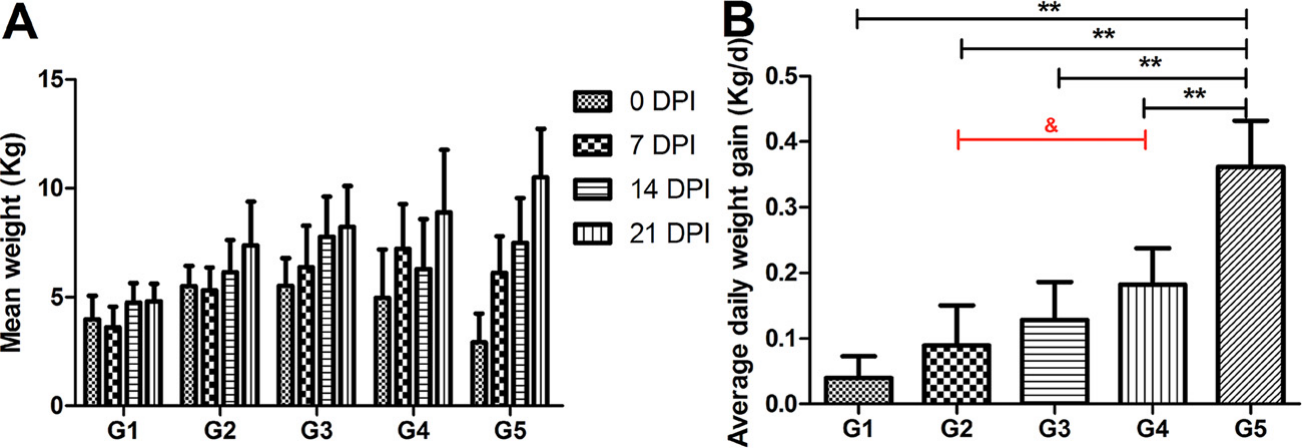
Analysis of body weight in different groups after infection. (A) Body weights were measured every 7 days from the day of inoculation (0 dpi) to the end of the experiment (21 dpi). (B) Average daily weight gain (ADWG) throughout the experimental period. Data are expressed as mean ± standard deviation (SD). *, *P* < 0.05 and **, *P* < 0.01 compared to G5; ^&^, *P* < 0.05, comparisons among G2, G3, and G4.

After bacterial challenge, the food intake of the pigs changed dramatically. The pigs infected with the CO strain via the IV+IP route (group 1 [G1]) exhibited depressed appetite from 2 days postinoculation (dpi), which was maintained until the day of necropsy with no obvious increase in body weight. The pigs in G1 had significantly lower body weights than those in the other four groups during the monitoring period (*P* < 0.01, Fig. 2A). Pigs infected with the NCO strain via the same route (G2) did not start to gain weight until 14 dpi. The weights of the pigs infected with the NCO strain via the IV+IP+IN (G3) or IN routes (G4) increased slightly during the experimental period, but less so than those of the pigs in the control group (G5).

As shown in Fig. 2B, the ADWG of pigs in all four challenge groups (G1 to G4) was significantly lower (*P* < 0.01) than that of the pigs in the control group (G5). The lowest ADWG was observed in G1 (CO, IV+IP), although there was no significant difference compared with G2 (NCO, IV+IP). Among groups infected with the NCO strain, the ADWG of G2 (IV+IP) was significantly lower than that of G4 (IN) (*P* < 0.05).

**FIG 3:**
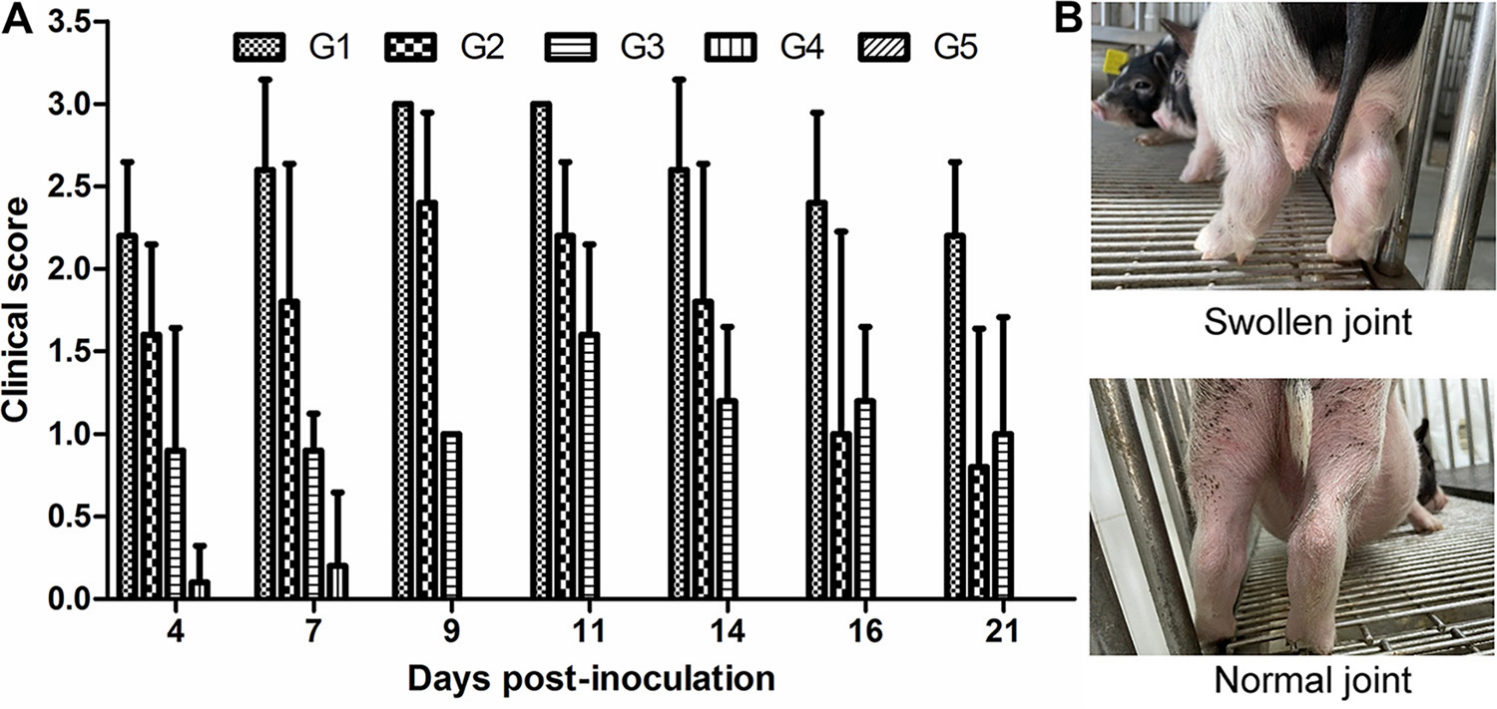
Clinical evaluation of arthritis. (A) Pigs were monitored and scored regularly after infection for joint swelling, lameness, and movement difficulties. Clinical scoring was performed as follows: 0 = no clinical signs of arthritis; 1 = one or more joints swollen slightly; 2 = two or more joints moderately swollen together with lameness and reluctance to move; 3 = two or more joints severely swollen together with severe lameness and difficulty moving. Data are expressed as mean ± SD. (B) Images of severely swollen joints (G1 group) and normal joints (G5 group).

Animals were monitored daily for general health and any clinical signs related to *M. hyorhinis* infection (Fig. 3A and B). Significant joint swelling and lameness were observed as early as 4 dpi in all animals in G1. Three of the pigs in G2 showed joint swelling and lameness at 4 dpi, while joint swelling was only slight in the other two. The symptoms of animals in G1 and G2 gradually worsened within 9 dpi. At 9 dpi, all the pigs in G1 and two pigs in G2 showed severe swelling in their joints, making movement difficult. The clinical symptoms began to alleviate around 11 dpi. The recovery of the animals in G2 was more significant than that of the animals in G1. At 21 dpi, only one pig had moderate joint swelling and mild lameness, one pig had slight joint swelling, and the clinical symptoms of the other pigs disappeared. However, in G1, all pigs had severe joint swelling and lameness, with one pig showing difficulty moving at 21 dpi. All animals in G3 showed slight joint swelling starting from 4 dpi until the end of the experiment. Three pigs showed short-term severe joint swelling with lameness, which disappeared quickly (within 2 to 6 days). Two animals in G4 developed very slight joint swelling at 4 and 7 dpi, and the symptoms had disappeared by 7 and 9 dpi, respectively. No clinical symptoms were observed in the control group (G5). With the exception of G4, all the infected groups had significantly higher clinical scores than the control group during the entire monitoring period (*P* < 0.01). There were significant differences (*P* < 0.01) between groups infected with different strains (G1 and G2) and between groups infected via different routes (G2, G3, and G4).

### Gross pathology at necropsy.

**FIG 4:**
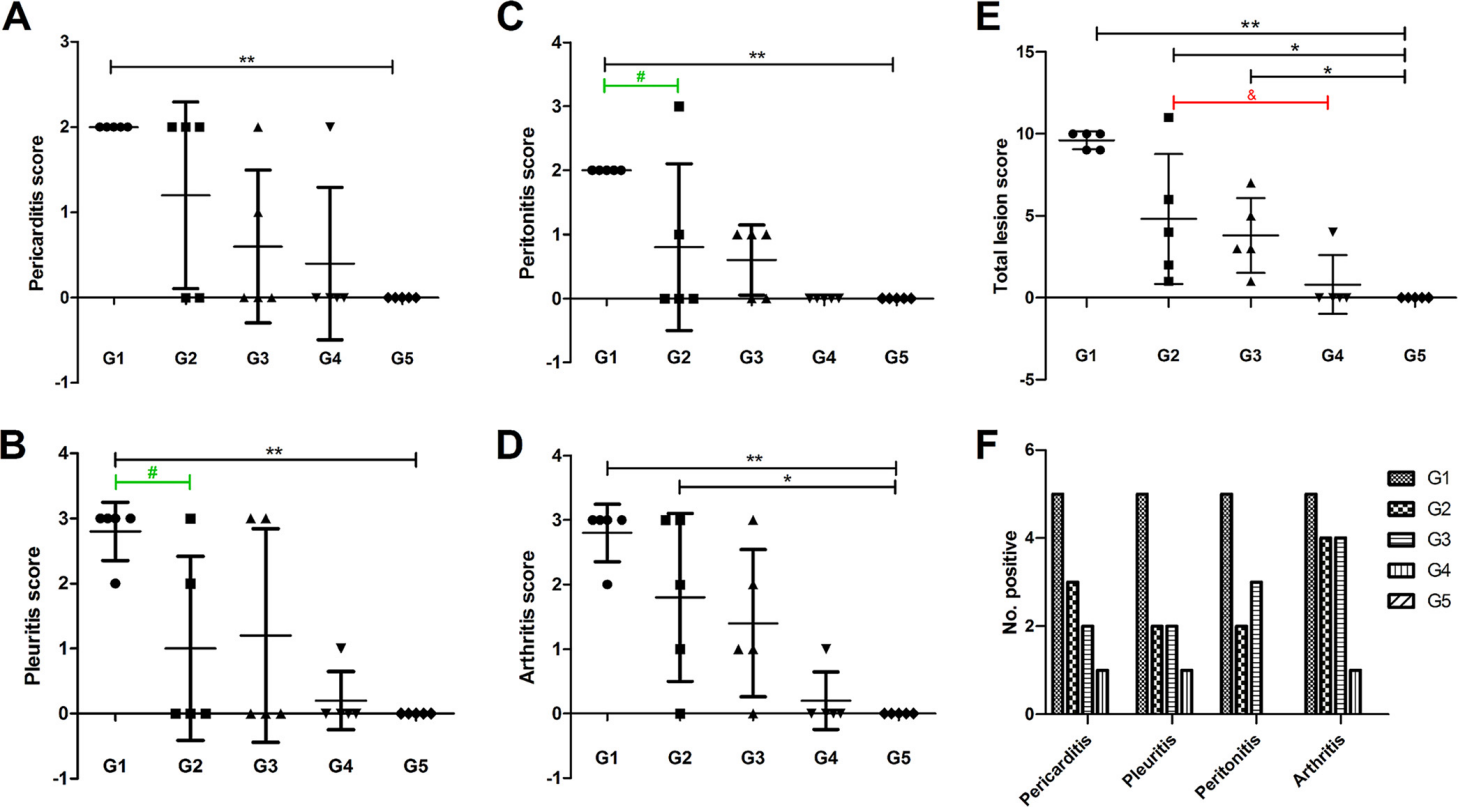
Polyserositis and arthritis lesion scores of the infected pigs. The pigs were euthanized at 21 dpi. (A-D) The development of pericarditis (A), pleuritis (B), peritonitis (C) and arthritis (D) were scored to reflect the relative severity according to previously defined criteria (14). (E) Total score was calculated as the sum of the scores of all four tissues. Data are expressed as the mean ± SD. *, *P* < 0.05 and **, *P* < 0.01, compared to G5; ^#^, *P* < 0.05, comparison between G1 and G2; ^&^, *P* < 0.05, comparisons among G2, G3, and G4. (F) The number of diseased pigs in each group was summarized.

On 21 dpi, all pigs were euthanized and necropsied. *M. hyorhinis*-induced pleuritis, pericarditis, peritonitis, and arthritis were observed and scored (Fig. 4A to D). All pigs in G1 exhibited moderate-to-severe pleuritis. More than 50% of the lung lobe adhered to the parietal pleura in these pigs, and adherence was also detected between lobes with adhesions over the lung surface. All pigs in G1 exhibited moderate pericarditis with diffuse inflammatory fibrinous adhesions between the heart and pericardium, significant thickening of the pericardium, and increased pericardial fluid accumulation in some pigs. All G1 pigs also exhibited diffuse moderate fibrinous peritonitis. All G1 pigs also developed moderate-to-severe arthritis, characterized mainly by marked edematous hypertrophy, increased turbid joint fluid, and suppurative foci in one or more joints. The number of diseased animals is summarized in Fig. 4F. Incidence of the four types of lesions in G1 all reached 100%, which was higher than that in the other four groups. One pig in G2 developed severe polyserositis (pleuritis, pericarditis, peritonitis) and arthritis, one developed moderate polyserositis and severe arthritis, one developed moderate polyserositis, and the other two developed mild-to-moderate arthritis. As shown in Fig. 4E, total scores in G1 and G2 were significantly increased compared to that in G5, for which no pathological changes were observed (*P* < 0.01 and *P* < 0.05, respectively). The total score for G1 (9.6 ± 0.55) was higher than that for G2 (4.8 ± 3.96), although the difference was not statistically significant (*P* = 0.201). The differences in pleuritis and peritonitis scores between G1 and G2 were significant (*P* < 0.05, Fig. 4B and C), indicating a difference in virulence between the two strains.

Two pigs in G3 showed moderate polyserositis with no/mild arthritis, two showed moderate-to-severe arthritis with no/mild peritonitis, and one showed only mild arthritis. The total score of G3 was significantly higher than that of the control group (*P* < 0.05, Fig. 4E). Only one pig in G4 developed mild polyserositis and arthritis, while no lesions were detected in the other four pigs. The total score of G4 was not significantly different from that of the control group (*P* = 0.644, Fig. 4E). Furthermore, among the groups inoculated with the NCO strain (G2, G3 and G4), the total score of G2 was significantly higher than that of G4 (*P* < 0.05, Fig. 4E).

Bacteria were re-isolated from tissue samples (tonsil, lung, heart, and joint fluid) of one pig with significant lesions each from G1 to G4, and of one from G5 as a negative control. *M. hyorhinis* was isolated from at least one tissue from each pig. Multilocus sequence typing (MLST) analysis showed that all isolates were the same ST as the strains used for infection.

### Detection of *M. hyorhinis*-specific antibodies.

**FIG 5:**
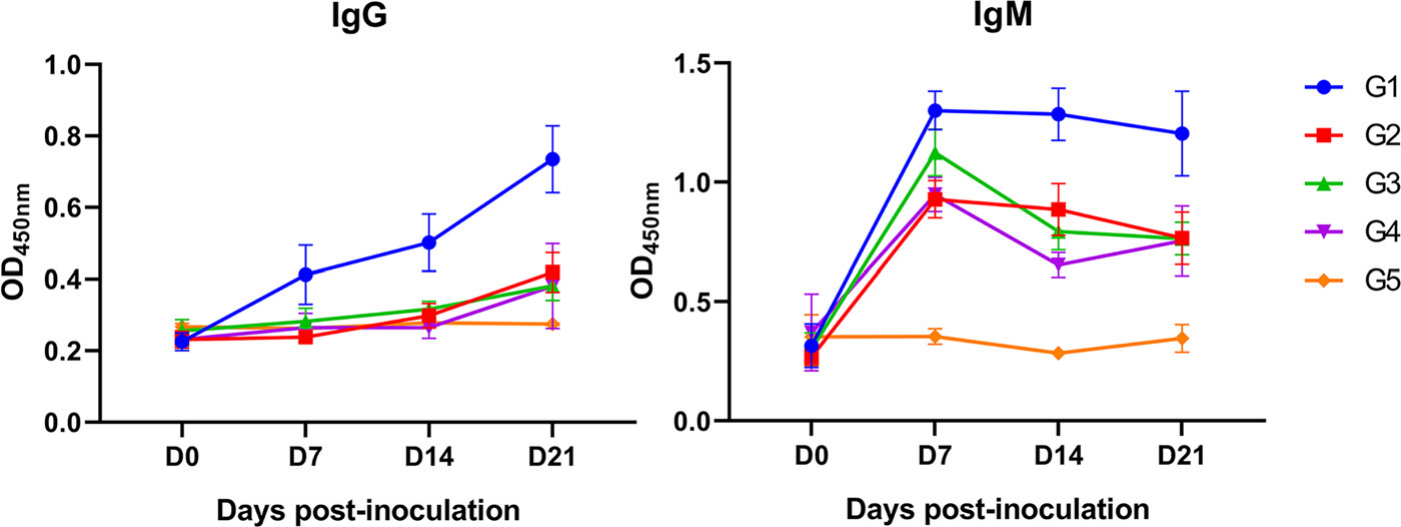
Serum IgG and IgM antibody titer in *M. hyorhinis*-infected pigs. After infection, serum samples were collected every 7 days and IgG and IgM antibody levels were detected by indirect enzyme-linked immunosorbent assay using *M. hyorhinis* whole cell protein as coating antigen. Data are expressed as mean ± SD.

IgG and IgM antibody production was measured every 7 days (Fig. 5). *M. hyorhinis*-specific IgG was detected in G1 at 7 dpi, and the titer increased significantly during the monitoring period. The IgG titer of G1 was significantly higher than those of the other groups (*P* < 0.01). IgG antibody responses in other infected groups were not detected until 21 dpi. The IgM response occurred earlier than the IgG response. The IgM titer of all infected pigs peaked at 7 dpi and decreased slowly thereafter. The IgM titer of the G1 pigs was significantly higher than those of the pigs in other groups (*P* < 0.01). No IgG or IgM response was detected in G5.

## DISCUSSION

The infection rate of *M. hyorhinis* is very high in farms all over the world. It has been widely recognized that *M. hyorhinis* infection can induce systemic inflammation, although most pigs do not develop disease. A key question is what determines the result of the occurrence of apparent diseases or an asymptomatic infection. The objective of this study was to evaluate the impacts of differences in the virulence of strains and of experimental systemic dissemination on the consequences of *M. hyorhinis* infection. Two *M. hyorhinis* strains, isolated from pigs with typical clinical symptoms (CO strain) and from clinical healthy asymptomatic pigs (NCO strain), were isolated and used to infect Bama miniature pigs. Consequently, compared with the NCO strain, the CO strain induced more serious body weight loss, joint swelling, lameness, and polyserositis, confirming the existence of differences in virulence between the two strains. Consistent with our results, previous studies have also indicated differences in the virulence of *M. hyorhinis* strains. Gois et al. (12) inoculated gnotobiotic piglets with three different strains isolated from pneumonic lungs of pigs via the IN route. One strain produced pneumonia alone, whereas the other two caused polyserositis, pericarditis, and arthritis in addition to pneumonia. Lin et al. (11) found that only pigs challenged with a mixture of three clinical isolated strains via intratracheal inoculation developed pneumonia (not typically observed during *M. hyorhinis* infection), while other animals infected with the ATCC strain (ATCC 27717) did not develop gross lung lesions and showed only minimally perivascular infiltration. A difference in tissue-specific tropism between strains were also implied in these studies, which was not observed between the two strains used in our study.

More importantly, we found that the NCO strain induced clinical and gross pathological changes in infected pigs, suggesting that strains isolated from asymptomatic pigs are not necessarily nonvirulent and may also induce disease under suitable conditions. Because *M. hyorhinis* is very common in pig farms, this result indicates that farms with *M. hyorhinis*-infected asymptomatic pigs should be alert to the possibility of *M. hyorhinis*-caused diseases. This also emphasizes the importance of implementing measures to control *M. hyorhinis* infection in pig farms to achieve long-term benefits. Naturally attenuated *M. hyorhinis* strains may also exist in the field, and further studies are required to investigate this issue.

The typical signs of disease associated with *M. hyorhinis* infection generally occur 3 to 10 days after exposure to the bacterium or the induction of a systemic reaction. Pigs may exhibit an elevated body temperature, apathy, loss of appetite, swollen joints, lameness, reluctance to move, and labored breathing. The acute clinical signs usually subside after 10 to 14 days. In case of arthritis, lameness and swollen joints may persist for 2 to 3 months (13, 14). In this study, the clinical signs of challenged animals began to resolve after 10 days postinoculation. The clinical scores for lameness and arthritis in G1, G2, and G3 pigs began to decrease after 11, 9, and 11 dpi, respectively. The recovery of animals inoculated with NCO strain in G2 was more significant than that of the animals inoculated with CO strain in G1. This may be due to the differences in the severity of the clinical diseases in these groups. The clinical signs of the animals in G4 group, which were very slight, disappeared after 7 dpi.

In this study, we investigated the influence of inoculation routes on the consequences of *M. hyorhinis* infection. Pigs inoculated with pathogens via systemic routes (IP and IV) showed typical clinical and pathological signs of diseases. However, those inoculated via the IN route did not show obvious diseases, except for a certain decrease in ADWG (Fig. 3 and 4). The increase in IgG and IgM antibody titers and the re-isolation of the bacteria postinfection confirmed successful infection in this group (Fig. 5). Similarly, Martinson et al. (15) found that a single IN administration of a *M. hyorhinis* preparation harvested from lysed-cell suspension after infection caused few/no signs of disease in cesarean-derived colostrum-deprived (CDCD) pigs. This may partly explain why many pigs do not show clinical symptoms (or maybe they are too slight to be noticed) when *M. hyorhinis* is widespread. It can be noted that the animals in G2 (IV 4.5 mL + IP 4.5 mL) were more severely affected than those in G3 (IV 3 mL+ IP 3 mL+ IN 3 mL). This difference could be attributed to the lower total amount of *M. hyorhinis* injected through the IV and IP routes in G3, due to the addition of the IN route. In contrast, IN inoculation of *M. hyorhinis* has also been reported to induce diseases, including pneumonia, polyarthritis, arthritis, and eustachitis (7, 12, 16, 17). In our study, animals were necropsied at 21 dpi. Neither significant clinical symptoms nor pathological changes were observed in the group of pigs inoculated via the IN route. However, it was unknown whether those pigs would show more obvious diseases after 21 dpi. Further studies are specially required to clarify the ability of *M. hyorhinis* to cause disease by means of nasal drops or aerosols, because these routes are very similar to field infection. It is important to know: (1) whether IN or aerosol infection of *M. hyorhinis* would cause diseases without any other factors; (2) would it finally cause a systemic dissemination; and (3) how does this dissemination happen? In our study, we did not observe differences in tissue tropism among the same strain inoculated through different routes. Pneumonia is not typical in *M. hyorhinis* infection. However, in some studies, the inoculation of *M. hyorhinis* induced pneumonia similar to that induced by M. hyopneumoniae, the pathogenesis of which is not yet clear. It seems from previous reports that pneumonia was more common when pigs were infected via IN or intratracheal routes (11, 12), which is contrary to our findings in this study. Whether tissue tropism is affected by infection route or strain characteristics needs more research for clarification.

The mechanisms responsible for systemic spread under field conditions are unclear. *M. hyorhinis* and *M. hyorhinis*-specific antibodies were more often detected in pigs infected with other pathogens, such as Haemophilus parasuis (18), porcine reproductive and respiratory syndrome virus (PRRSV) (6, 18), porcine circovirus 2 (PCV2) (19), or M. hyopneumoniae (19–21). The presence of *M. hyorhinis* in the serous cavities was always accompanied by a diagnosis of other disease conditions, mainly those of the respiratory tract (22). It is assumed that infection by other pathogens (19) may cause lung tissue damage and help *M. hyorhinis* to overcome the physiological barrier and spread to other tissues. In an investigation of the function of the nasal microbiota on subsequent systemic *M. hyorhinis* infection, Blanco-Fuertes et al. (23) observed that specific amplicon sequence variants of pathogens found only in farms with *M. hyorhinis*-induced diseases were not shared by farms with other diseases. In addition to coinfection, it is possible that other factors, such as stress, may also promote the systemic spread of *M. hyorhinis*. Kinne et al. (7) reported that standard thermometric stress (swim-test) enhanced the incidence and intensity of pneumonia induced by IN inoculation of *M. hyorhinis*. Many invasive bacteria utilize the host plasminogen/plasmin system to disseminate from their initial colonization site and gain entry into distal tissue sites by secreting plasminogen activators or expressing plasminogen receptors on their surface (24). Our previous study confirmed the ability of *M. hyorhinis* to hijack plasminogen through surface-exposed proteins (8). Further studies are required to investigate possible differences in this hijacking ability between strains, and whether such differences correlate positively with strain pathogenicity.

Differences in virulence and tissue tropism between strains have been observed. A convenient way to determine or predict these differences would be valuable. Genotyping is a useful tool to differentiate strains, monitor infections, and conduct epidemiological investigations. MLST and multilocus variable-number tandem-repeat analysis (MLVA) techniques have been developed for *M. hyorhinis* genotyping (25, 26). Genotyping studies have indicated that *M. hyorhinis* strains exhibit a high degree of diversity between areas and farms, and even within a single farm (25, 27). Unfortunately, no connection has been identified yet between any genotype and the virulence characteristics of strains (28). New genotyping techniques have been established in recent years that utilize additional proteins or proteins which are more sensitive to microenvironment and evolution pressures (26, 28, 29). With the continuous development of genotyping methods, connections between them strains might be found in the future.

The Bama pigs used in the current study are a minipig breed developed naturally in the Chinese remote mountainous areas of Bama County (Guangxi Zhuang Autonomous Region). Bama minipigs are considered suitable laboratory animals for their genetic stability, high level of inbreeding, and small bodies. Consequently, these animals are inexpensive to feed and easy to handle. The clinical and pathological symptoms of Bama pigs after challenge were very typical, suggesting that this breed is a good animal model for studies of *M. hyorhinis* vaccines, therapies, and pathogenic mechanisms.

There are still some limitations of this study and questions that have not been fully answered. The major ones are listed as follows. (i) Most of the pigs infected with *M. hyorhinis* showed no apparent clinical disease. One main purpose of this research was to investigate which factor determines the results of *M. hyorhinis* infection. Therefore, the NCO strain was chosen to compare different routes, which may be more representative of the clinical infections on most farms. These results can help us answer whether there is a potential risk of significant diseases with “harmless” infections of this type of strain. However, a comparison over different inoculation routes with the CO strain was not included in this study, since neither obvious clinical nor pathological changes were observed in pigs which received only IN inoculation of the NCO strain. Further studies could be carried out to explore whether IN infection of a high-virulence strain, such as the CO strain, would induce notable clinical diseases. (ii) In this study, each pig received a total dose of 10^9^ color-changing units (CCU) of *M. hyorhinis*. In previous studies, doses of 1 × 10^9^ to 5 × 10^9^ CCU per pig were usually used to develop a challenge model of *M. hyorhinis* (11, 30–32). To our knowledge, the minimum doses for artificial infection via different routes are unknown, and the number of *M. hyorhinis* required in the natural infection process is also unclear. A dose of 10^9^ CCU might be higher than that in natural infection, and the potential difference in infection results should be considered. However, the bacterial number required for artificial infection may be higher than that required for natural infection, because repeated exposure to infected pigs usually happens under natural conditions.

### Conclusion.

*M. hyorhinis* strains were isolated from a pig with typical clinical symptoms of *M. hyorhinis* infection and from an asymptomatic pig. Both strains induced clinical and pathological changes in pigs after inoculation via the combined IP and IV route. The disease induced by the CO strain was more severe than that induced by the NCO strain. Comparison of infection consequences with the NCO strain via different routes revealed that the combined IP and IV route induced the most serious disease. Thus, our findings suggest that both the virulence of strain and systemic dissemination affect the consequences of *M. hyorhinis* infection.

## MATERIALS AND METHODS

### Mycoplasma isolation.

Pigs were obtained from two commercial farms. Farm 1 had a known history of problems with polyserositis and arthritis, which were determined based on necropsy results. *M. hyorhinis* infection was confirmed by nested PCR (described below) of nasal swab samples from 55 pigs, which showed a positive ratio of 72.7%. One positive, 6-week-old Duroc-Landrace-Yorkshire pig from this farm, with swollen joints and severe diffuse fibrinous peritonitis, was used for pathogen isolation. Farm 2 had very stable animal health, without any respiratory problems, arthritis, or behavioral disorders based on long-term slaughter examination and clinical history. However, 58.9% of nasal swab samples from 56 pigs tested positive for *M. hyorhinis* by nested PCR analysis. One positive, 8-week-old Duroc-Landrace-Yorkshire pig from this farm was used for pathogen isolation, and no gross pathological changes were observed at necropsy.

For *M. hyorhinis* isolation, palatine tonsil (hereinafter referred to as “tonsil”), lung, heart, and joint tissues (swabs) were sampled from each pig. Tissues were rinsed three times with sterile phosphate-buffered saline (PBS) and then soaked in 10 mL KM2 medium (a modified Friis medium). The tissues were then cut into small pieces (approximately 1 to 3 mm^2^). The suspensions were shaken for 1 min before the supernatant was carefully aspirated and filtered (0.45-μm pore). Finally, the filtrates were diluted (10×) with KM2 medium and cultured at 37°C. Cultures were checked daily and subcultured when the red color of the medium changed to yellow. Mycoplasma species were identified by PCR. After three passages, the cultures were plated on solid KM2 medium. A single colony was picked, cultured with liquid medium, and confirmed again by PCR.

### *M. hyorhinis*-specific PCR.

Total DNA was extracted from the mycoplasma isolates and nasal swab samples using the Bacterial DNA Kit (Omega Bio-Tek, USA) according to the manufacturers’ instructions. All samples were identified by PCR targeting the *p37* gene of *M. hyorhinis*. The isolated pathogens were identified by regular PCR. The PCR mix (total volume 25 μL) contained 10 pmol of each primer, 2 × *Taq* Master Mix (Vazyme, China), and 2 μL DNA template. Amplification reactions were performed in a thermocycler under the following conditions: denaturation at 95°C for 5 min followed by 30 cycles of 95°C for 1 min, 52°C for 1 min, and 72°C for 1 min, with a final extension at 72°C for 10 min. To improve detection sensitivity, a nested PCR strategy was used to detect *M. hyorhinis* in nasal swab samples. After the amplification described above, inner primers were added and the product of the first amplification reaction was used as the template for the second-round PCR. The second-round amplification was performed under the following conditions: denaturation at 95°C for 5 min followed by 30 cycles of 94°C for 30 s, 54°C for 30 s, and 72°C for 30 s, with a final extension at 72°C for 10 min. Primer sequences are listed in Table 1.

**TABLE 1:** Primer information

Primer	Sequence	Product length (bp)
Outer primers		857
F:	5′-TAATTGGGATTGGATTGCTCT-3′	
R:	5′-TGCTTGGCCTACAATACTTAT-3′	
Inner primers		352
F:	5′-CAATGAATTCCGCTTCGTTTT-3′	
R:	5′-CTAATAAAGACCCGCCAAGTG-3′	

### Multilocus sequence typing.

The isolated *M. hyorhinis* strains were cultured in KM2 medium until the medium color changed from red to yellow. Cells were collected by centrifugation at 15,000 × *g* for 20 min, resuspended in 70 μL double-distilled water (ddH_2_O), and boiled for 15 min. The crude lysate samples were stored at −20°C prior to testing. Six *M. hyorhinis* housekeeping genes (*dnaA*, *rpoB*, *gyrB*, *gltX*, *adk*, and *gmk*) were selected for multilocus sequence typing using previously described primers and procedures (25). The PCR products were sequenced and submitted to the PubMLST database (www.pubmlst.org) to assign allele and ST numbers. Sequences that did not match any of the existing ST types were registered as novel.

### Experimental design.

Bama miniature pigs (aged 6 weeks) were obtained from a pig farm originally developed from cesarean-derived colostrum-deprived pigs. The pigs from this farm were negative for PRRSV, pseudorabies virus, PCV2, classical swine fever virus, african swine fever virus, M. hyopneumoniae infection, and M. hyorhinis infection based on the routine serological tests and PCR tests performed every 6 months. Twenty-five pigs were picked and confirmed to be free from *M. hyorhinis* colonization by nested PCR of nasal swab samples. Animals were randomly separated into five groups (*n* = 5 per group) (Table 2). Animals in groups 1 to 4 were inoculated with 10^9^ CCU of *M. hyorhinis* suspended in 9 mL PBS, equally divided over the different inoculation routes in groups 1 to 3. One strain isolated from the symptomatic pig and another strain from the asymptomatic pig were used in challenge experiments via different inoculation routes (in a mostly combined way), including intravenous, intraperitoneal, and intranasal. Details of the *M. hyorhinis* strains, inoculation volumes, and routes are shown in Table 2. Pigs from different groups were housed in individual pens with metal slatted flooring and metal gated sides and provided with water and a commercial pelleted diet free from antibiotics. The groups challenged with different strains and the negative-control group were kept in separate rooms with independent ventilating systems.

**TABLE 2:** Design of the challenge experiment

Group	Challenge	Strain source	Inoculation method
G1	Challenged	Symptomatic pig	IV (4.5 mL) + IP (4.5 mL)
G2	Challenged	Asymptomatic pig	IV (4.5 mL) + IP (4.5 mL)
G3	Challenged	Asymptomatic pig	IV (3 mL)+ IP (3 mL)+ IN (3 mL)
G4	Challenged	Asymptomatic pig	IN (9 mL in total, twice, with 15-min interval)
G5	Not challenged	NA^*a*^	NA

a NA, not applicable.

### Clinical evaluation.

After infection, pigs were monitored daily for clinical signs, including elevated body temperature, apathy, loss of appetite, swollen joints, lameness, and labored breathing. Mean body weights were measured on days 0, 7, 14, and 21. Average daily weight gain during the entire experimental period was calculated and lameness and arthritis were scored as previously described (30): 0 = no clinical signs of arthritis; 1 = one or more joints slightly swollen; 2 = two or more joints moderately swollen together with lameness and reluctance to move; 3 = two or more joints severely swollen together with severe lameness and difficulty in moving. The observer was blinded to inoculation status.

### Necropsy and pathological examination.

The piglets were euthanized at 21 days postinfection, and a gross pathological examination was performed. The thoracic and abdominal cavities were exposed and examined for signs of polyserositis (pleuritis, pericarditis, and peritonitis). The tarsal and carpal joints were exposed and examined for arthritis. Pleuritis, pericarditis, peritonitis, and arthritis were scored separately to reflect relative severity according to previously defined criteria (30), and a total score was calculated as the sum of the scores for the four tissues. Scoring was carried out by observer blinded to inoculation status.

For one pig in each group, bacteria were re-isolated from tonsil, lung, heart, and joint tissues (swabs) to exclude undetected infection with other mycoplasmas or other *M. hyorhinis* strains that occurred prior to the experiment or unexpected infection from the environment during the experimental period. PCR and MLST analysis were carried out to confirm the species and strains.

### Serology test.

*M. hyorhinis*-specific serum IgG and IgM antibodies were evaluated every 7 days postinoculation using an in-house indirect enzyme-linked immunosorbent assay (ELISA). Briefly, *M. hyorhinis* cells were collected by centrifugation at 15,000 × g for 20 min at 4°C, washed, and resuspended in PBS. After lysis by ultrasonication, the supernatant was collected, and the protein concentration was determined using a bicinchoninic acid (BCA) assay kit (Beyotime, China) according to the manufacturer’s instructions. The supernatant was diluted to 5 μg/mL with carbonate-bicarbonate buffer (pH 9.6) and used to coat 96-well ELISA plates overnight at 4°C. After blocking with 5% bovine serum albumin (BSA), each well was incubated with 100 μL of serum sample (1:100 in PBS containing 5% BSA) for 30 min at 37°C, followed by 100 μL horseradish peroxidase (HRP)-conjugated goat anti-swine IgG (Bethyl Laboratory, USA)/IgM (Bio-Rad, USA) (1:10,000 in PBS containing 5% BSA). After washing, the reaction was visualized by incubation with tetramethylbenzidine-hydrogen peroxide (TMB; Beyotime, China) substrate for 10 min at room temperature. The optical density (OD) of the solution was measured at 450 nm using a BioTek uQuant microplate reader (Bio-Tek, Winooski, VT, USA).

### Statistical analysis.

Data were expressed as the mean ± standard deviation (SD). ADWG data were analyzed using a one-way analysis of variance (ANOVA) with *post hoc* Bonferroni comparison for selected pairs. For pathological evaluation at necropsy, data were analyzed using the Kruskal–Wallis test with a Dunn’s *post hoc* test for selected pairs. Repeated measures ANOVA were used to analyze changes in body weight, clinical symptoms, and antibody production during the experimental period. *P* < 0.05 was set as the threshold for statistical significance.

### Ethics approval.

The animal experimental procedures conformed to the guidelines of Jiangsu Province Animal Regulations (Government Decree No. 45). The animals in this study were under ethical approval by the Committee on the Ethics of Animal Experiments in Jiangsu Academy of Agricultural Sciences (Protocolnumber PDC 2021001). All efforts were made to minimize animal suffering in experiments.

### Data availability.

The data sets supporting the conclusions of this article are included within the article and its additional files.
